# Comparison of Cabergoline and Quinagolide in Prevention of Severe Ovarian Hyperstimulation Syndrome among Patients Undergoing Intracytoplasmic Sperm Injection

**DOI:** 10.22074/ijfs.2018.5259

**Published:** 2018-01-15

**Authors:** Robabeh Taheripanah, Mahshid Vasef, Marzieh Zamaniyan, Anahita Taheripanah

**Affiliations:** 1 Infertility and Reproductive Health Research Center, Shahid Beheshti University of Medical Sciences, Tehran, Iran; 2 Infertility Center, Department of Obstetrics and Gynecology, Mazandaran University of Medical Sciences, Sari, Iran; 3 Diabetes Research Center, Mazandaran University of Medical Sciences, Sari, Iran; 4 Department of Molecular and Cellular Sciences, Faculty of Advanced Sciences and Technology Pharmaceutical Sciences Branch, Islamic Azad University, Tehran, Iran

**Keywords:** Dopamine Agonists, Dopamine D2, Ovarian Hyperstimulation Syndrome, Receptors

## Abstract

**Background:**

The aim of the current study is to compare quinagolide with cabergoline in prevention of ovarian hyperstimulation syndrome (OHSS) among high risk women undergoing intracytoplasmic sperm injection (ICSI).

**Materials and Methods:**

This randomized clinical trial study was performed from March 2015 to February 2017. One hundred and twenty six women undergoing ICSI who were at high risk of developing OHSS (having over 20 follicles of >12 mm), were randomized into two groups. The first group received cabergoline 0.5 mg and the second group received quinagolide 75 mg every day for 7 days commencing on the day of gonadotropin-releasing hormone (GnRH) agonist administration. Then OHSS symptoms as well as their severity were assessed according to standard definition, 3 and 6 days after GnRH agonist administration. Ascites were determined by trans-vaginal ultrasound. Other secondary points were the number of oocytes and the number of embryos and their quality. Quantitative and qualitative data were analyzed using Student’s t test, and Chi-square or fisher’s exact test, respectively. A P<0.05 was considered statistically significant.

**Results:**

The incidence of severe OHSS in the quinagolide-treated group was 3.1% while it was 15.8% in cabergolinetreated subjects (P<0.001). Ascites were less frequent after treatment with Quinagolide as compared to cabergoline (21.9 vs. 61.9%, respectively) (P=0.0001). There was no significant statistical deferences between the two groups in terms of mean age, number of oocytes, metaphase I and metaphase II oocytes, and germinal vesicles. There was a significant difference between cabergoline and quinagolide groups regarding the embryo number (P=0.037) with cabergoline-treated group showing a higher number of embryos. But, the number of good quality embryo in quinagolide-treated individuals was significantly higher than that of the cabergoline-treated group (P=0.001).

**Conclusion:**

Quinagolide seems to be more effective than Cabergoline in prevention of OHSS in high-risk patients undergoing ICSI (Registration number: IRCT2016053128187N1).

## Introduction

Ovarian hyperstimulation syndrome (OHSS) could be a life-threatening complication of assisted reproduction treatment (ART) (1). The incidence of OHSS varies between 6 and 12% based on the studied population and classification of disease; also, severe cases have an incidence of 2-4% (2, 3). OHSS is characterized by the presence of multiple luteinized cysts within the ovaries that induce ovarian enlargement and increase capillary permeability with enhanced fluid shift to the third space (4). Recent findings have introduced vascular endothelial growth factor (VEGF) as the mainstay for increased capillary permeability (2, 5). OHSS has a broad spectrum of clinical manifestations ranging from mild to severe symptom. Subjects with mild disease presented with enlargement of ovaries, lower abdominal pain and discomfort, temporary nausea and vomiting, diarrhea, and abdominal distention. Persistent toxic symptoms or the presence of ascites indicates a progressive OHSS that requires treatment (3, 6, 7). 

Raised serum estradiol levels to concentrations of >2,500 pg/mL, and observations of large numbers of small and intermediate-sized ovarian follicles, are signs of high risk necessitating to proceed with great caution (8, 9). Administration of cabergoline, a dopamine agonist as a prophylactic agent is associated with significant reductions in the incidence of symptoms and signs of moderate to severe OHSS. This drug inhibits vascular endothelial growth factor 2 phosphorylation (VEGFR-2) (9-12) and decreases the incidence of OHSS and cycle cancellation rate without having any adverse effects on gestation. Quinagolide (Norprolac™) is a non-ergot extract and dopamine agonist with a chemical structure similar to apomorphine. Binding of quinagolide to D2 dopamine receptors on the lactotroph cells in the anterior pituitary decreases adenylyl cyclase activity, reduces the intracellular cyclic adenosine monophosphate, and inhibits prolactin excretion (13). The specificity of quinagolide for D2-type dopamine receptors diminishes its side effects compared to dopamine agonists (6, 13, 14).

Several studies have indicated that quinagolide effectively reduces the development of OHSS (6, 15). Therefore, the aim of the present study was to compare the quinagolide and cabergoline effects in preventing severe OHSS in high risk female patients who undergo intracytoplasmic sperm injection (ICSI), and to evaluate quinagolide’s effect on the oocyte and embryo quality.

## Materials and Methods

The present study was a parallel single-blind randomized clinical trial (IRCT2016053128187N1) with a 1:1 allocation ratio, recruiting 126 patients, who had undergone assisted reproductive procedure and were at risk of severe OHSS. The patients were randomly allocated to one of the study groups according to a random allocation sequence generated by a statistician using a computer software. The sequence was built through generating block size of 4.

The study was conducted in Infertility and Reproductive Health Research Center and Imam Hussein Medical Center, Shahid Beheshti University of Medical Sciences, Tehran, Iran, from March 2015 to February 2017. The project was approved by the Ethics Committee (IR.SBMU. RETECH.REC.1395.542) and institutional review board of Shahid Beheshti University of Medical Sciences, Tehran, Iran, and it was initiated after obtaining written informed consents from all participants. Randomization on the day of gonadotropin-releasing hormone (GnRH) agonist administration was based on a computer-generated random list which determined the random allocation of the subjects into the two groups.

**Fig.1:**
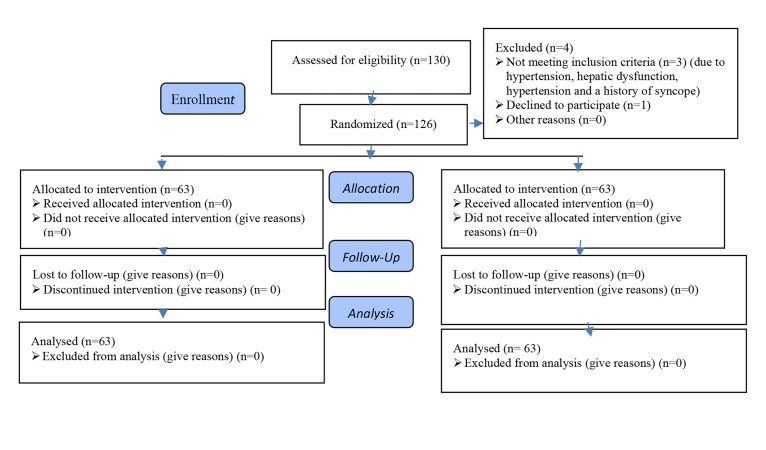
Flowchart of the trial.

Selection and randomization of the patients were performed by a nurse, using a series of sequentially numbered sealed envelopes; therefore, the sequence of allocation was hidden. The study was single-blinded, because the physicians were blind to the treatment group, but the patients were aware of the management option (Fig .1).

In this study, patients of 20-40 years old, who had 20 oocytes and serum estradiol levels of >3000 pg/ml on the day of GnRH agonist injection during ICSI cycles, were recruited. The inclusion criteria were being at high risk of developing OHSS and not having hepatic dysfunction, hypertension and a history of syncope. All participants underwent controlled ovarian hyperstimulation (COH) with gonadotropin/GnRH-antagonist protocol. Ovarian stimulation using recombinant-follicle-stimulating hormone (FSH, GONAL-f, Serono, Switzerland) was started on day 3 of cycle at a dose of 150 IU per day. 

Transvaginal ultrasound was performed every 3 days to examine the follicular development. Also, serum estradiol levels were measured every 2-3 days using radioimmunoassay method. After 5 days of stimulation, when at least two follicles with diameters of 14 mm were observed, GnRH antagonist (Cetrotide, Merk, USA) or (Orgalutran, Organon, the Netherlands) was started with a daily dose of 0.25 mg until administration of GnRH agonist. Final oocyte maturation was triggered when at least two follicles with diameters of at least 17 mm were observed, using a single intramuscular injection of 0.2 mg GnRH agonist (Decapeptyl, Ferring GmbH, Germany). Oocytes were collected 36-38 hours later using transvaginal- guided follicle aspiration. All embryos were frozen after fertilization through ICSI. On day of GnRH agonist administration, patients were randomized using computer- generated random tables into two groups.

The first group comprised of 63 women, was treated with 0.5 mg cabergoline (Dostinex™, Pfizer, USA) every day for 7 days and the second group comprised of 63 women, was treated with quinagolide 75 mg (Norprolac™, Ferring, Denmark) every day for 7 days. Diagnosis of OHSS as well as determination of its severity was performed according to Golan’s classification (16), on days 3 and 6 after GnRH agonist administration. The patients vital signs and weight were recorded at each visit. Transvaginal ultrasound was used to measure the ovarian volume and estimate the volume of pelvic free fluid. Data were extracted from the Checklist, clinical and laboratory notes and ultrasound reports. Age, body mass index (BMI), number of retrieved oocytes, number of metaphase . and II oocytes and germinal vesicles, number of embryos and number of high quality embryos were all recorded in specified data sheet. All patients were checked for any related symptoms or side effects of cabergoline and quinagolide.

### Statistical analysis

This was a randomized clinical trial study. To detect 20% difference in OHSS rates that is considered significant (1, 2) with a power of 80% and a=0.05, 63 patients in each group were needed. Statistical analysis was done using SPSS 21.0 (SPSS Inc., Chicago, IL USA). Effect size for comparing two means was determined by computing the mean difference between the two groups, and then dividing the result by the pooled standard deviation, according to Cohen’s d effect size. So, it is likely to have a negative effect size. However, if just the magnitude was important, we could take the absolute difference so that the effect size would be positive (17). Quantitative data were presented as mean ± SD. Quantitative and qualitative data were analyzed using Student’s t test, and Chi- square or fisher’s exact test, respectively. A P<0.05 was considered statistically significant.

## Results

A total of 130 women were recruited into the study. Four women were omitted from the research due to various reasons including declining to participate, having hypertension, hepatic dysfunction, or history of syncope, and discontinuing the treatment or loss to follow-up (Fig .1). The mean age of the patients in cabergoline and quinagolide groups were 31.05 ± 5.2 and 31.63 ± 4.4 years old, respectively. There was no significant differences between the mean ages of the two groups. Also, the two groups were not significantly different in terms of other major demographic characteristics such as type of infertility, menstrual cycle pattern, BMI and duration of infertility (Table 1). There was significant differences between quinagolide and cabergoline groups regarding the incidence of OHSS (22.2 vs. 47.6%, respectively) (P=0.001).

**Table 1:** Clinical and hormonal characteristics of patients in two groups of patients entering the study


Variable	Quinagolide n=63	Cabergoline n=63	P value

Age (Y)	31.63 ± 5.2	31.05 ± 4.4	0.503
Body mass index (BMI)	26.4 ± 3.8	27.5 ± 3.2	0.174
Type of infertility			
Primary	20 (31.7)	21 (33.3)	0.762
Secondary	10 (15.8)	11 (17.46)	0.762
Cause of infertility			
Female factor	3 (4.76)	2 (3.17)	0.644
Male factor	11 (17.46)	10 (15.87)	0.644
Both (male+PCOS)	4 (6.34)	5 (7.93)	0.644
Unexplained	1 (1.58)	2 (3.17)	0.644


Data are presented as mean + SD or n (%). PCOS; Polycystic ovary syndrome.

**Table 2:** The outcomes of ovarian stimulation in quinagolide and cabergoline-treated groups


Variable	Quinagolide n=63	Cabergoline n=63	Effect (95% CI)	P value

E2 on day of GnRH agonist (pg/ml)	3293.74 ± 3836.9	3615.79 ± 1473.5	0.11 (-0.24-0.46)	0.304
HB (g/dl)	12.10 ± 1.42	12.61 ± 1.17	-0.39 (-0.74-0.04)	0.034
HCT	36.85 ± 4.38	38.37 ± 3.77	-0.37 (-0.72-0.02)	0.045
Number of oocytes retrieval	29.02 ± 11.45	28.76 ± 6.46	0.03 (-0.32-0.38)	0.443
Number of GV	4.02 ± 2.93	3.60 ± 2.38	0.16 (-0.19-0.51)	0.834
Number of MI	2.81 ± 1.92	3.49 ± 2.15	-0.33 (-0.68-0.02)	0.786
Number of MII	22.58 ± 9.57	22.05 ± 7.88	0.06 (-0.29-0.41)	0.386
Number of embryo	6.22 ± 15.00	5.59 ± 17.23	-0.38 (-0.73-0.02)	0.037
Number of high quality of embryos	18.3 ± 5.1	14 ± 8.6	0.61 (0.25-0.96)	0.001
OHSS	14 (22.2)	30 (47.6)	0.46 (0.27-0.79)	0.001
Mild	6 (9.5)	9 (14.28)	0.66 (0.25-1.76)	0.432
Moderate	6 (9.5)	11 (17.46)	0.54 (0.22-1.38)	0.545
Severe	2 (3.1)	10 (15.8)	0.2 (0.04-0.87)	0.001
GI symptoms	38 (59.4)	40 (63.5)	0.95 (0.72-1.25)	0.857
Ascites	14 (21.9)	39 (61.9)	0.36 (0.22-0.59)	0.0001
Paracentesis	7 (10.9)	17 (27.0)	0.41 (0.18-0.92)	0.021
Admission	2 (3.1)	14 (22.2)	0.14 (0.03-0.60)	0.001


Data are presented as mean + SD or n (%). E2; Estradiol, GnRH; Gonadotropin releasing hormone, HB; Hemoglobin, HCT; Hematocrit, GV; Germinal vesicle, MI; Metaphase I, MII; Metaphase II, OHSS; Ovarian hyper stimulation syndrome, GI; Gastrointestinal, and CI; Confidence interval.l.

The incidence of severe OHSS was considerably lower in the Quinagolide group (3.1% in quinagolide-treated group vs. 15.8% in the cabergoline-treated group, P<0.001). Ascites were less frequent after treatment with quinagolide as compared to cabergoline (21.9 vs. 61.9%, P=0.0001). Also, ascites paracentesis was significantly lower in quinagolide group compared to cabergoline group (10.9 and 27%, respectively, P=0.021). Hematocrit and hemoglobin were significantly lower after treatment with quinagolide as compared to cabergoline (P=0.045 and 0.034, respectively) and admission rate was significantly lower in quinagolide group compared to cabergoline (3.1 vs. 22.2%, P=0.001, Table 2). There was no statistically significant deferences between the two groups in terms of gastrointestinal symptoms, estradiol levels on the day of agonist administration, the number of oocytes, metaphase I and metaphase II oocytes and germinal vesicles. The number of embryos in cabergoline group was significantly higher in comparison to the quinagolide group (17.23 vs. 15.00%, P=0.037), but the number of good quality embryos in quinagolide group was significantly higher than the cabergoline group (P=0.001, Table 2).

## Discussion

OHSS is a life-threatening complication induced by ART which is more frequently observed when a strong ovarian response occurs (1). This strong ovarian response is characterized by development of several ovarian follicles and high levels of serum estradiol (2, 4). Prophylactic administration of cabergoline and quinagolide as dopamine agonists, is associated with a significant decrease in incidence of signs and symptoms related to moderate or severe OHSS (1, 6).

This prospective randomized study showed that risk of OHSS is more markedly reduced following administration of quinagolide at a dose of 75 mg compared to cabergoline at a dose of 0.5 mg among high risk patients. In our study, the incidence of severe OHSS in quinagolide-treated group was significantly lower compared to that of cabergoline- treated group. Kamel et al. (18) compared quinagolide 75 mg with cabergoline 0.5 mg in prevention of OHSS among high-risk patients undergoing *in vitro* fertilization (IVF). 

Patients received drugs for 8 days starting from the day of human chorionic gonadotropin injection. The number of patients who developed OHSS was similar in the two groups, which was not consistent with our findings. Busso et al. (2) in a randomized double-blind placebo-controlled trial, evaluated different doses of quinagolide in prevention of early OHSS. Their findings showed that quinagolide when given at three dose (50, 100, 200 mg/day), was effective in reducing the incidence of moderate and severe OHSS from 4-12% to 0-2. Their results were similar to ours regarding the incidence of moderate OHSS after prophylactic administration of quinagolide at the dose of 200 mg/day and severe OHSS at the dose of 50 mg/day. 

According to our results, the number of patients with ultrasound evidence of ascites within the 6 days after GnRH agonist administration, was significantly reduced in quinagolide compared to cabergoline-treated group. Similarly, Baumgarten et al. (6) in a randomized controlled prospective study on role of quinagolide in preventing OHSS among high risk ICSI patients, showed that the number of patients with ultrasound evidence of ascites within the initial 8 days after human chorionic gonadotropin (hCG) administration, was significantly lower in quinagolide- treated group than control group.

In our study, admission rate was significantly reduced in quinagolide-treated group as compared to cabergoline- treated group. Kamel et al. (18) found that hospitalization rate is similar in cabergoline and quinagolide-treated groups which was contrary to our results.

We found no significant statistical differences between the two groups in terms of the number of oocytes, metaphase I and metaphase II oocytes, and germinal vesicles. Although the number of embryos in cabergoline-treated group was significantly higher compared to quinagolide- treated group, the number of good quality embryos in quinagolide- treated group was significantly higher than that of the cabergoline-treated group. Kiliç et al. (19) evaluated the effects of cabergoline in prevention of OHSS in women at risk undergoing IVF treatment cycles and showed that in cabergoline-treated group, total number of embryos, number of total good quality embryos, and the fertilization rate were significantly higher than control group. In this study, cabergoline and quinagolide administration had no negative impact on oocyte and embryos numbers and their quality which was consistent to previously published data.

In our study, there was no statistically significant differences were observed between the two groups in terms of gastrointestinal symptoms. Busso et al. (2) noted that upper gastrointestinal symptoms, especially nausea and vomiting, were more frequent following administration of quinagolide compared to placebo, especially when quinagolide was given at high doses. One important limitation of the present study was the small sample size. In this regard, the small number of patients restricts the generalizability of the results of the present study. Advanced trials with adjusted doses are therefore required. There was also some potential sources of bias including interactions with other drugs.

## Conclusion

Quinagolide seems to be more effective than cabergoline in preventing OHSS among high-risk patients undergoing ICSI. Further studies should be performed to compare quinagolide and cabergoline to achieve a firm conclusion.
